# Supplementary material for: Availability of Hib, Pneumococcal, Rotavirus, and HPV vaccines in China: Implication for equity in access to immunization services
Source: PLOS Glob Public Health. 2026 Apr 21;6(4):e0006151. doi: 10.1371/journal.pgph.0006151 (PMC13098980; doi:10.1371/journal.pgph.0006151)
Supplement: S1 Appendix — Table A. Vaccine availability at clinic level, 2022 (n, %). Table B. Absolute and relative inequality on vaccine availability, 2022. Table C. Classification of economic regions in China. Table D. Data collection table - free vaccines included in the National Immunization Program (NIP), 2022. Table E. Data collection table - self-paid vaccines (non-NIP vaccines), 2022. (DOCX) [file pgph.0006151.s001.docx]

Availability of Hib, Pneumococcal, Rotavirus, and HPV vaccines in China: Implication for equity in access to immunization services

**Supporting Information**

**Table A.** Vaccine availability at clinic level, 2022 (n, %)

|  | **Number of vaccination clinics providing following vaccines** | | | | **Total** |
| --- | --- | --- | --- | --- | --- |
|  | **Hib** | **Pneumococcal** | **Rotavirus** | **HPV** |  |
| **Total** | 1704 (65.3%) | 1259 (48.3%) | 1534 (58.8%) | 1823 (69.9%) | 2609 |
| **Economic regions** |  |  |  |  |  |
| Eastern | 777 (78.8%) | 620 (62.9%) | 684 (69.4%) | 816 (82.8%) | 986 |
| Central | 491 (67.6%) | 369 (50.8%) | 453 (62.4%) | 487 (67.1%) | 726 |
| Western | 436 (48.6%) | 270 (30.1%) | 397 (44.3%) | 520 (58.0%) | 897 |
| **Urban-Rural Location** |  |  |  |  |  |
| Urban | 519 (89.6%) | 509 (87.9%) | 519 (89.6%) | 525 (90.7%) | 579 |
| Rural | 1185 (58.4%) | 750 (36.9%) | 1015 (50.0%) | 1298 (63.9%) | 2030 |

Note: This table presents the number and proportion of vaccination clinics in the sample that offer specific types of vaccines among all clinics providing National Immunization Program (NIP) vaccines for children (N = 2,609).

**Table B.**  Absolute and relative inequality on vaccine availability, 2022

| **Vaccine** | **SII** | **Std. error** | **p-value** | **CI** | **Std. error** | **p-value** |
| --- | --- | --- | --- | --- | --- | --- |
| Hib | 1.306 | 0.734 | 0.087 | 0.209 | 0.022 | 0.000 |
| MenAC-Hib | -0.766 | 0.377 | 0.067 | -0.008 | 0.005 | 0.130 |
| DTaP-Hib | 1.442 | 0.560 | 0.016 | 0.147 | 0.020 | 0.000 |
| DTaP-IPV-Hib | 3.648 | 0.614 | 0.000 | 0.408 | 0.019 | 0.000 |
| LLR | 1.906 | 0.447 | 0.000 | 0.230 | 0.022 | 0.000 |
| RV5 | 2.508 | 0.432 | 0.000 | 0.343 | 0.021 | 0.000 |
| HPV2 | 1.950 | 0.446 | 0.000 | 0.213 | 0.022 | 0.000 |
| HPV4 | 2.924 | 0.532 | 0.000 | 0.314 | 0.022 | 0.000 |
| HPV9 | 2.504 | 0.561 | 0.000 | 0.311 | 0.022 | 0.000 |
| PCV13 | 2.964 | 0.381 | 0.000 | 0.362 | 0.021 | 0.000 |

Note: This table presents both the slope index of inequality (SII) and the concentration index (CI) for the availability of selected vaccines. The SII measures the absolute difference in vaccine availability between the most and least economically advantaged groups. A higher absolute value indicates a greater disparity. The CI reflects the relative distribution of vaccine availability across the economic spectrum: a larger positive value indicates greater availability in economically better-off areas, while a larger negative value indicates greater availability in economically disadvantaged areas.

**Table C**. Classification of economic regions in China

| **Region** | **Number of Provinces** | **Provinces Included** | **Total GDP in 2022 (CNY trillion)** | **Total Population (million)** | **GDP per capita (CNY thousand)** |
| --- | --- | --- | --- | --- | --- |
| Eastern | 11 | Beijing, Tianjin, Hebei, Shanghai, Jiangsu, Zhejiang, Fujian, Shandong, Guangdong, Hainan, Liaoning | 67.0 | 607.7 | 110.2 |
| Central | 8 | Shanxi, Anhui, Jiangxi, Henan, Hubei, Hunan, Heilongjiang, Jilin | 29.2 | 419.0 | 69.8 |
| Western | 12 | Inner Mongolia, Guangxi, Chongqing, Sichuan, Guizhou, Yunnan, Xizang, Shaanxi, Gansu, Qinghai, Ningxia, Xinjiang | 26.1 | 383.1 | 68.1 |
| **Total** | **31** | — | **122.3** | **1,409.8** | **86.7** |

Note: Hong Kong SAR, Macao SAR, and Taiwan are not included in this regional classification in this manuscript.

**Table D.** Data collection table - free vaccines included in the National Immunization Program (NIP), 2022

| **Vaccine** | **Number of doses administered** | **Notes** |
| --- | --- | --- |
| **Total** |  |  |
| Hepatitis B vaccine |  |  |
| Hepatitis B immunoglobulin |  |  |
| Poliomyelitis vaccine |  |  |
| Bacillus Calmette–Guérin (BCG) vaccine |  |  |
| DTaP vaccine (diphtheria, tetanus, and acellular pertussis) |  |  |
| DT vaccine (diphtheria and tetanus) |  |  |
| Measles–Rubella vaccine (MR) |  |  |
| Measles–Mumps–Rubella vaccine (MMR) |  |  |
| Measles vaccine |  |  |
| Measles–Mumps vaccine (MM) |  |  |
| Japanese encephalitis vaccine, live attenuated |  |  |
| Meningococcal vaccine (Group A) |  |  |
| Meningococcal vaccine (Group A + C) |  |  |
| Hepatitis A vaccine, live attenuated |  |  |
| Hemorrhagic fever vaccine (bivalent) |  |  |
| Anthrax vaccine |  |  |
| Leptospirosis vaccine |  |  |
| Japanese encephalitis vaccine, inactivated |  |  |
| Hepatitis A vaccine, inactivated |  |  |
| COVID-19 vaccine |  |  |
| Varicella vaccine |  |  |
| Neonatal immunoglobulin |  |  |
| Influenza vaccine |  |  |
| Hib vaccine |  |  |
| Pneumococcal vaccine |  |  |
| Mumps vaccine |  |  |
| Other (please specify in Notes) |  |  |

Note: This table presents the structure of the reporting form used for collecting information on free vaccines included in the National Immunization Program in 2021. The original form was presented as a webpage in Chinese.

**Table E.** Data collection table - self-paid vaccines (non-NIP vaccines), 2022

| **Vaccine** | **Number of doses administered** | **Price per dose (CNY)** | **Notes** |
| --- | --- | --- | --- |
| **Total** | — | — |  |
| Hepatitis A vaccine |  |  |  |
| Hepatitis B vaccine |  |  |  |
| Hepatitis B immunoglobulin |  |  |  |
| Japanese encephalitis vaccine |  |  |  |
| Rabies vaccine |  |  |  |
| Rabies immunoglobulin |  |  |  |
| Mumps vaccine |  |  |  |
| Hib vaccine |  |  |  |
| Domestic vaccine |  |  |  |
| Imported vaccine |  |  |  |
| Pneumococcal polysaccharide vaccine (23-valent) |  |  |  |
| Domestic vaccine |  |  |  |
| Imported vaccine |  |  |  |
| Varicella vaccine |  |  |  |
| Rotavirus vaccine |  |  |  |
| Domestic monovalent vaccine |  |  |  |
| Imported pentavalent vaccine |  |  |  |
| Inactivated poliovirus vaccine (IPV) |  |  |  |
| Influenza vaccine |  |  |  |
| Trivalent domestic vaccine for 6 months–3 years (0.25 ml) |  |  |  |
| Trivalent imported vaccine for 6 months–3 years (0.25 ml) |  |  |  |
| Trivalent domestic vaccine for ≥3 years and adults (0.5 ml) |  |  |  |
| Trivalent imported vaccine for ≥3 years and adults (0.5 ml) |  |  |  |
| Quadrivalent domestic vaccine for 6 months–3 years (0.25 ml) |  |  |  |
| Quadrivalent imported vaccine for 6 months–3 years (0.25 ml) |  |  |  |
| Quadrivalent domestic vaccine for ≥3 years and adults (0.5 ml) |  |  |  |
| Quadrivalent imported vaccine for ≥3 years and adults (0.5 ml) |  |  |  |
| Live attenuated intranasal influenza vaccine |  |  |  |
| Tetanus antitoxin |  |  |  |
| Tetanus immunoglobulin |  |  |  |
| Tick-borne encephalitis vaccine |  |  |  |
| Human papillomavirus (HPV) vaccine |  |  |  |
| Bivalent vaccine |  |  |  |
| Quadrivalent vaccine |  |  |  |
| 9-valent vaccine |  |  |  |
| Pneumococcal conjugate vaccine (13-valent) |  |  |  |
| Domestic vaccine |  |  |  |
| Imported vaccine |  |  |  |
| Meningococcal vaccine (ACYW135) |  |  |  |
| Meningococcal conjugate vaccine (AC) |  |  |  |
| Measles–Mumps–Rubella vaccine (MMR) |  |  |  |
| Mumps vaccine |  |  |  |
| Meningococcal AC–Hib combined vaccine |  |  |  |
| Quadrivalent combination vaccine |  |  |  |
| Pentavalent combination vaccine |  |  |  |
| Enterovirus 71 (EV71) vaccine |  |  |  |
| COVID-19 vaccine |  |  |  |
| Other (please specify in Notes) |  |  |  |

Note: This table presents the structure of the reporting form used for collecting information on self-paid vaccines (non-NIP vaccines) in 2021. The original form was presented as a webpage in Chinese.
