# Supplementary material for: Availability of Hib, Pneumococcal, Rotavirus, and HPV vaccines in China: Implication for equity in access to immunization services
Source: PLOS Glob Public Health. 2026 Apr 21;6(4):e0006151. doi: 10.1371/journal.pgph.0006151 (PMC13098980; doi:10.1371/journal.pgph.0006151)
Supplement: Attachment — Submitted filename: 20260305.Response_to_comments.docx [file pgph.0006151.s003.docx]

**Authors’ Response to Editorial and Reviewers’ Comments**

**Reference number**
PGPH-D-25-02255

**Title**
Availability of Hib, Pneumococcal, Rotavirus, and HPV vaccines in China: Implication for equity in access to immunization services

**Date**
2026-03-08

| **No.** | **Comments** | **Responses** |
| --- | --- | --- |
|  | **EDITOR** |  |
| 1 | Line 101-105: Add details regarding sampling framework. | Thank you for this important suggestion. We agree that clarification of the sampling framework is essential for understanding the representativeness of the data source. We have now added a dedicated subsection titled “Sampling framework” in the Methods section to describe the nationally representative, stratified multi-stage sampling design used in the 2022 National Health Accounts institutional survey.  Although the present study primarily used data from primary health care facilities, the survey was conducted within the broader National Health Accounts sampling framework. Therefore, for completeness and transparency, we have described the full hierarchical sampling process across provincial, prefecture, and county levels, including the selection principles for public health institutions, medical institutions, and primary care facilities.  The revised manuscript now includes detailed information on stratification by administrative level, the selection of at least one-third of prefectures within provinces, the requirement that sampled counties account for no less than 15% of all counties, and the sampling approach for community health centers and township health centers. These additions can be found in Lines 114-142.  These revisions improve methodological transparency and allow readers to better assess the representativeness of the study sample. |
| 2 | Line 125-127: Clarify if 15 centres that did not complete the vaccine data form differed from the 355 districts that were included for analysis. | Thank you for this helpful suggestion. We examined whether the 15 excluded centers differed systematically from those included in the final analysis. The excluded centers were distributed across 12 provinces and comprised seven counties, seven urban districts, and one county-level city. We compared geographic distribution and administrative level between excluded and included centers and did not observe statistically significant differences.  We have now clarified this in the manuscript as follows:  “A total of 15 centers did not complete the vaccine data form and were excluded from the analysis. Among the excluded centers, seven were located in counties, seven in urban districts, and one in a county-level city, and they were distributed across 12 provinces. No significant differences were observed between excluded and included centers in terms of geographic distribution, administrative level, or key institutional characteristics.  The corresponding revision has been made in Lines 160–164. |
| 3 | Line 128-135: Clarify if 2,824 public primary health facilities that had data extracted differed from 2,423 who did not. Also how other exclusions may have affected results. | Thank you for this important comment. The 2,423 facilities were excluded because they were not public primary health care institutions, in accordance with the predefined study scope and inclusion criteria. Our study specifically focused on public primary health care facilities that provide vaccination services; therefore, these facilities were not eligible for inclusion rather than excluded due to missing data. As such, a direct comparison between these two groups is not applicable.  We have clarified this point in the revised manuscript by adding the following sentence in Lines 166–167: “We excluded 2,423 health facilities that were not public primary health care facilities.” |
| 4 | Line 136-138: Add references for databases | Thank you for this suggestion. We have added the appropriate reference citation for the database used. Specifically, we have cited the National Bureau of Statistics of China National Data platform as follows:  *National Bureau of Statistics of China. National Data. [cited 27 Feb 2026]. Available from: https://data.stats.gov.cn/english/*  The reference has been added to the revised manuscript as reference 13. |
| 5 | Line 208 Results: Is there any data for vaccine coverage (i.e. how many children received vaccines) in each of the regions. | Thank you for this important question. The dataset used in this study does not support the calculation of vaccine coverage. Estimating coverage requires individual-level data and an appropriate population denominator, such as the total number of eligible children in each region, which were not available in the National Health Accounts institutional survey.  Our analysis is based on institutional-level administrative data on vaccine procurement and reporting. Therefore, the study focuses on vaccine availability at the regional level, reflecting the presence and supply of vaccines across areas rather than individual uptake or coverage. Vaccine coverage and vaccine availability represent related but conceptually distinct dimensions of immunization system performance. |
| 6 | Line 517-518: Specify the wealth status of the three economic regions. | Thank you for this helpful suggestion. We have now specified the economic status of the three regions by adding information on total GDP, total population, and GDP per capita for 2022. These indicators provide a clearer description of the relative economic development levels of the eastern, central, and western regions. The eastern region has the highest GDP per capita, followed by the central and western regions, reflecting the commonly recognized economic gradient across these regions. The corresponding information has been added in the Supporting Information Table S3. |
| 7 | Correct minor spelling and grammatical errors. | Thank you for the suggestion. Minor spelling and grammatical errors have been corrected throughout the manuscript. |
|  | **JOURNAL REQUIREMENTS** |  |
| 8 | 1. Please amend your detailed Financial Disclosure statement. This is published with the article. It must therefore be completed in full sentences and contain the exact wording you wish to be published.  i. Please clarify all sources of financial support for your study. List the grants, grant numbers, and organizations that funded your study, including funding received from your institution. Please note that suppliers of material support, including research materials, should be recognized in the Acknowledgements section rather than in the Financial Disclosure.  ii. State the initials, alongside each funding source, of each author to receive each grant. For example: "This work was supported by the National Institutes of Health (####### to AM; ###### to CJ) and the National Science Foundation (###### to AM)." iii. State what role the funders took in the study. If the funders had no role in your study, please state: “The funders had no role in study design, data collection and analysis, decision to publish, or preparation of the manuscript.” iv. If any authors received a salary from any of your funders, please state which authors and which funders. | Thank you for this important reminder. We have carefully revised the Financial Disclosure statement to ensure that it is written in full sentences and reflects the exact wording intended for publication. |
| 9 | 2. Please ensure that your Ethics Statement is available in its entirety at the beginning of your Methods section, under a subheading 'Ethics Statement'. | Thank you for this helpful reminder. We have revised the manuscript to ensure that the full Ethics Statement now appears at the beginning of the Methods section under the subheading “Ethics Statement,” in accordance with the journal’s requirements. The statement is presented in its entirety as requested. |
| 10 | 3. Please upload separate figure files in .tif or .eps format. Also, remove the figures from your manuscript file but keep the legends.  For more information about figure files please see our guidelines:  <https://journals.plos.org/globalpublichealth/s/figures>  <https://journals.plos.org/globalpublichealth/s/figures#loc-file-requirements>  4. We notice that your supplementary tables are included in the manuscript file. Please remove them and upload them with the file type 'Supporting Information'. Please ensure that each Supporting Information file has a legend listed in the manuscript after the references list. | Thank you for your guidance regarding file formatting.  All figures have now been uploaded as separate high-resolution .tif files in accordance with the journal’s requirements. The figures have been removed from the main manuscript file, while all figure legends have been retained.  All supplementary tables have been removed from the manuscript and uploaded separately as Supporting Information files. We have also added a “Supporting Information” section after the References list in the manuscript, with complete legends corresponding to each file.  We appreciate the opportunity to revise the submission to ensure full compliance with the journal’s formatting guidelines. |
| 11 | 5. We note that you have indicated that there are restrictions to data sharing for this study. PLOS only allows data to be available upon request if there are legal or ethical restrictions on sharing data publicly. For more information on unacceptable data access restrictions, please see <http://journals.plos.org/plosone/s/data-availability#loc-unacceptable-data-access-restrictions>.  Before we proceed with your manuscript, please address the following prompts:  a)If there are ethical or legal restrictions on sharing a de-identified data set, please explain them in detail (e.g., data contain potentially identifying or sensitive patient information, data are owned by a third-party organization, etc.) and who has imposed them (e.g., a Research Ethics Committee or Institutional Review Board, etc.). Please also provide contact information for a data access committee, ethics committee, or other institutional body to which data requests may be sent.  b) If there are no restrictions, please upload the minimal anonymized data set necessary to replicate your study findings to a stable, public repository and provide us with the relevant URLs, DOIs, or accession numbers. For a list of recommended repositories, please see  https://journals.plos.org/plosone/s/recommended-repositories. You also have the option of uploading the data as Supporting Information files, but we would recommend depositing data directly to a data repository if possible. | Thank you for your inquiry regarding data sharing.  The dataset used in this study was obtained from the China National Health Development Research Center (NHDRC) and is part of the National Health Accounts administrative reporting system. The data are owned and managed by the relevant governmental authority.  There are formal legal and administrative restrictions on public data sharing. Specifically:   - According to the “Administrative Measures for the Management of National Health Financial Annual Report Data (全国卫生健康财务年报数据管理办法)” issued by the National Health Commission, data may not be publicly released, transmitted via the internet, or provided externally without authorization from the Department of Finance of the National Health Commission. - In addition, Article 22 of the “Interim Measures for the Management of Data Security and Personal Information Protection in Medical and Health Institutions (关于印发医疗卫生机构数据安全和个人信息保护管理办法(试行)的通知)” (国卫规划发[2026] No. 6) stipulates that medical and health institution data may not be provided externally without formal approval from the authorized institutional leadership or designated data governance authority.   Under these regulations, the authors are not permitted to publicly deposit the dataset in a repository.  Researchers who wish to access the data may submit a formal request to the China National Health Development Research Center, which manages the dataset and oversees data access approvals. Data access is subject to institutional review and authorization in accordance with national regulations.  We appreciate the opportunity to clarify this matter. |
|  | **REVIEW COMMENTS TO THE AUTHOR** |  |
| 12 | Reviewer #1: This is a very interesting paper but a few pieces of additional information would make it stronger they include: | We sincerely thank the reviewer for the positive and constructive comments on our manuscript. We greatly appreciate the insightful suggestions, which have helped us improve the quality and clarity of the paper. |
| 13 | 1. Foregrounding the prices earlier in the paper AND stating whether the clinic or district needs to outlay money to purchase a stock. To do so, I would add a table of the prices of the vaccines earlier on, and in that table i would clarify 1. Hib (a,b,c,d types), 2 PCV (a.b.c etc.) so that when the reader is looking at the data it is clear how much financial burden is not only on the family but on the clinics themselves. Because you state the the demand side analysis has been done, it should be cited more clearly. Similarly, i would add the give the population # and eligible population # for each region (children under 5, girls 11 etc.) as well, if possible as the average household or per capita income of the regions in question.  2. If possible, document the differential health budgets for each region or district (per capita if possible) | Thank you for this thoughtful suggestion. We did not incorporate vaccine price comparisons into the Results section because the primary objective of this study is to assess regional patterns of vaccine availability, rather than to evaluate determinants of demand or price responsiveness.  Price was not included as an explanatory variable in our analytical framework, and the study design does not support causal inference regarding the relationship between price and availability. Presenting price differences within the Results could unintentionally imply that price is a tested driver of the observed disparities. Including such comparisons without formally modeling their association with availability may risk overinterpretation.  To provide appropriate contextual background while preserving the analytical focus of the study, we have summarized regional population size, and GDP per capita in Supplementary Table S3. These data are intended to aid interpretation rather than serve as explanatory variables in the main analysis.  We believe this approach maintains conceptual clarity while addressing the reviewer’s concern. |
| 14 | 3. Explaining more clearly the difference between Table 1--which shows rates of 80s-90s % for Hib, and Table 2 which shows 50-70% These two tables seem to contradict one another. | We thank the reviewer for highlighting this important point. The apparent discrepancy arises from the different units of analysis used in the two tables. Table 1 presents vaccine availability at the district or county level, defined as the presence of at least one clinic within the district or county providing the vaccine. In contrast, Table 2 presents availability at the individual clinic level, defined as the proportion of sampled clinics that provided the vaccine.  Because a district or county is classified as having vaccine availability in Table 1 if at least one clinic provides the vaccine, it is not necessary that all clinics within that district or county offer the vaccine. As a result, clinic level availability percentages shown in Table 2 are lower. The two tables therefore reflect different analytical levels and are complementary rather than contradictory.  To improve clarity, we have added a note below Table 2 explicitly explaining this distinction:  *“Note: Table 1 reports availability at the district or county level, defined as the presence of at least one clinic providing the vaccine. Table 2 reports availability at the clinic level. Because not all clinics within a district or county necessarily offer the vaccine, clinic-level percentages are lower and the two tables reflect different units of analysis.”* |
| 15 | 4. In the study limitations, please include the limits of description of vaccine in the binary fashion you chose (present (even one dose) or absent) this likely under estimates chronic shortages and intermittent stock outs which can drive down demand. | We thank the reviewer for this important and insightful comment. We agree that defining vaccine availability in a binary manner may obscure temporal fluctuations in supply and potentially underestimate chronic shortages or intermittent stock outs.  In the revised manuscript, we have expanded the Limitations section to clarify that vaccine availability was operationalized as present if at least one dose was administered during the study year and absent otherwise. We now explicitly acknowledge that this annual binary classification does not capture within-year variation in supply. Clinics experiencing prolonged shortages or recurrent short-term stock interruptions may still have been classified as having availability if any doses were administered, which could overestimate sustained access. Conversely, temporary administrative or procurement delays may not be fully reflected in the measure.  We have added text to explain that this limitation may affect the precision of estimating supply stability and continuity, and that future studies using longitudinal inventory or stock management data would be better positioned to assess chronic and intermittent shortages.  Specifically, we added in Line 435-440:  *“…In addition, this binary classification does not capture temporal variations in supply within the year. It may underestimate chronic shortages or intermittent stock outs. Clinics experiencing prolonged supply constraints over an extended period may still have been classified as having availability if any doses were administered during the year. Similarly, short term stock interruptions that temporarily restricted access would not be reflected in the annual binary measure…”* |
| 16 | Reviewer #2: The investigators report an ERC-exempt, Gates Foundation-funded study of the availability at the clinic and county level of four non-program vaccines in China that WHO recommends that all national immunization programs include – HPV, PCV, Hib, and rotavirus vaccines. The study was based on analysis of 2022 survey (the China National Health Accounts Study) conducted by the China National Health Development Research Center, a non-governmental technical center affiliated with the National Health Commission (China’s MoH) that provides data for policy makers. The study purpose is “… to investigate regional and urban-rural disparities in vaccine availability across China.” They found that there were significant disparities in the availability of the four non-program vaccines at the clinic level and at the county level, with availability relate to socioeconomic development level. Availability was positively associated with wealth of the jurisdiction and was greater in urban compared with rural settings. They concluded that their findings highlight “significant inequities in the availability of WHO-recommended non-NIP vaccines across China, particularly affecting rural and economically disadvantaged regions,” and that “the highest inequalities were observed for higher-priced vaccines such as DTaP-IPV-Hib and PCV13.” They recommend that “… appropriate policy should be developed to prioritize expanding vaccine provision in underserved areas, strengthening rural service capacity, and considering the inclusion of these vaccines in the NIP (the National Immunization Program).”  Vaccines only work when used, and availability is essential for using a vaccine. As the authors state, the four study vaccines are recommended for all countries. Thus, the topic is important. The methods for assessing availability were appropriate, within the constraints of their data source. The figures and tables are excellent. Their conclusions were supported by the data presented, and their recommendations also follow from the data presented.  I have only a few suggestions to improve this very good manuscript. | We sincerely thank the reviewer for the thoughtful, balanced, and highly encouraging assessment of our manuscript. We are particularly grateful for the careful summary of our study objectives, methods, and key findings, which reflects a deep and accurate understanding of our work.  We greatly appreciate the reviewer’s recognition of the importance of the topic, the appropriateness of our methodological approach within the constraints of the available data, and the strength of the figures, tables, and conclusions. The positive evaluation of the study’s contribution to understanding inequities in the availability of WHO-recommended non-NIP vaccines is especially encouraging.  We are also thankful for the constructive suggestions provided to further strengthen the manuscript, and we have carefully addressed each of them in the revised version below. |
| 17 | Readers will appreciate more information about the actual survey that they used in their analysis. The reader will benefit from knowing what the China National Health Development Research Center is (e.g., government, independent, or government-affiliated), and the survey methods, ideally including response rate (if applicable). Currently, the manuscript’s description of the methods is made by referral to reference 13, which is an independent analysis published in 2020 of an “adolescent health expenditure in this study covered the period from 1st January to 31st December 2014,” quoting reference 13. This survey was in 9 provinces, but the present (2022) survey was in 27 provinces. Perhaps the authors could provide a brief paragraph on the present survey’s sampling strategy and sampling results. The reader would also benefit from being able to view the survey instrument (which had been augmented for vaccine access) in supplementary materials (or a link to the survey instrument in the references). | Thank you for this constructive suggestion. We agree that providing a clearer description of the current survey enhances methodological transparency.  In the revised manuscript, we have removed the reference to the 2014 publication and now present a direct description of the 2022 survey conducted by the China National Health Development Research Center. The Methods section has been expanded to include the sampling framework and facility selection procedures. The survey questionnaire is also included in the Supporting Information S4 Table and S5 Table.  These revisions are intended to ensure that readers can fully understand the survey design and data collection process while maintaining clarity and focus in the main text. |
| 18 | Lines 73-75 – The sentence about program vaccines states that the vaccines that are included in the National Immunization Program “… are provided free of charge to children under six years old.” The upper age limit of the program was extended to 18 years of age before 2021. For example, the schedule states that “children below 18 years of age who have not completed their NIP vaccination series by the recommended ages should be caught up based on the following principles …” (National Health Commission of The People's Republic of China. Childhood Immunization Schedule for National Immunization Program Vaccines - China (Version 2021). China CDC Wkly. 2021 Dec 24;3(52):1101-1108. doi: 10.46234/ccdcw2021.270). The authors could indicate that during the study period (2022), there were no program vaccines routinely recommended beyond 6 years of age, but catch-up vaccination is free of charge through 18 years. | Thank you for highlighting this important and timely policy update. We agree that the recent incorporation of the bivalent HPV vaccine into China’s National Immunization Program for 13-year-old females is highly relevant to the interpretation of our findings.  In response to your suggestion, we have revised the *Limitations* section to explicitly note that, since the completion of our study, the bivalent HPV vaccine has been included in the National Immunization Program. We also clarify that our findings reflect the policy context of 2022 and may not fully capture subsequent national-level expansions in availability. The relevant reference has been added accordingly.  We appreciate the reviewer’s careful attention to evolving policy developments, which strengthens the contextual relevance of the manuscript. |
| 19 | During the study period, there were no HPV vaccines licensed for males in China. The authors should indicate that all HPV vaccine availability measures in the present study pertained to females only. | Thank you for this important clarification. During the study period in 2022, HPV vaccines were licensed only for females in China. To avoid any ambiguity regarding the target population, we have now added a clarifying statement in the Materials and Methods section (Vaccines subsection) specifying that all HPV vaccine availability measures in this study pertain to female vaccination services only in line 195-196:  *“…During the study period in 2022, HPV vaccines were licensed only for females in China; therefore, all HPV vaccine availability measures in this study pertain to female vaccination services…”*  We appreciate the reviewer’s attention to this regulatory context, which strengthens the clarity and precision of the manuscript. |
| 20 | In the limitations section, the authors mention that “Rapid policy changes further complicate the study’s relevance, as the data may not reflect current conditions. Several provinces have recently integrated the HPV vaccine into their local immunization programs, likely enhancing its availability beyond what is represented in this study.” The authors should indicate that HPV2 vaccine is now included in the National Immunization Program for 13-year-old females (Wang F, Cao L, Li K, et al. Technical and Implementation Guidelines for the Introduction of Human Papillomavirus Vaccine into China's National Immunization Program. China CDC Wkly. 2025 Dec 12;7(50):1545-1548. doi: 10.46234/ccdcw2025.262). | Thank you for highlighting this important and timely policy development. We agree that the recent inclusion of the bivalent HPV vaccine into China’s National Immunization Program for 13-year-old females is highly relevant to the interpretation of our findings.  In response to your suggestion, we have revised the *Limitations* section to explicitly reflect this update. The manuscript now states:  *“In addition, since the completion of this study, the bivalent HPV vaccine has been incorporated into China’s National Immunization Program for 13-year-old females, which is expected to substantially expand its nationwide availability [26]. Therefore, the findings presented here should be interpreted within the policy context of 2022 and may not fully reflect subsequent national-level expansions.”*  We have also added the corresponding reference as suggested. We appreciate the reviewer’s attention to evolving national immunization policy, which improves the contextual accuracy and timeliness of our manuscript. |
| 21 | I was unable to follow the link in reference 12, receiving a 404 response. Authors should check to verify access to that website. | Thank you for pointing this out. We have verified that the DOI <https://doi.org/10.1787/9789264270985-en> correctly resolves to the OECD publication *A System of Health Accounts 2011: Revised Edition* (OECD, Eurostat, World Health Organization, 2017). The publication is available on the official OECD site, where both the abstract and PDF download are accessible, and is also archived in WHO’s repository.  If it doesn’t work, please try this link <https://www.oecd.org/en/publications/a-system-of-health-accounts-2011_9789264270985-en.html> |
|  | **FIGURE RESUBMISSIONS** |  |
| 22 | While revising your submission, we strongly recommend that you use PLOS’s NAAS tool (<https://ngplosjournals.pagemajik.ai/artanalysis>) to test your figure files. NAAS can convert your figure files to the TIFF file type and meet basic requirements (such as print size, resolution), or provide you with a report on issues that do not meet our requirements and that NAAS cannot fix.  After uploading your figures to PLOS’s NAAS tool - <https://ngplosjournals.pagemajik.ai/artanalysis>, NAAS will process the files provided and display the results in the "Uploaded Files" section of the page as the processing is complete. If the uploaded figures meet our requirements (or NAAS is able to fix the files to meet our requirements), the figure will be marked as "fixed" above. If NAAS is unable to fix the files, a red "failed" label will appear above. When NAAS has confirmed that the figure files meet our requirements, please download the file via the download option, and include these NAAS processed figure files when submitting your revised manuscript. | Thank you for the recommendation to use the PLOS NAAS tool.  All figure files have now been uploaded to the NAAS system for verification. The files were processed and confirmed to meet the journal’s technical requirements. We have downloaded the NAAS-processed TIFF files and included them as separate figure files in the revised submission.  We appreciate the guidance in ensuring full compliance with the journal’s figure formatting standards. |
